# Magnetic hyperthermia enhance the treatment efficacy of peri-implant osteomyelitis

**DOI:** 10.1186/s12879-017-2621-4

**Published:** 2017-07-25

**Authors:** Chih-Hsiang Fang, Pei-I Tsai, Shu-Wei Huang, Jui-Sheng Sun, Jenny Zwei-Chieng Chang, Hsin-Hsin Shen, San-Yuan Chen, Feng Huei Lin, Lih-Tao Hsu, Yen-Chun Chen

**Affiliations:** 1 Institute of Biomedical Engineering, National Taiwan University, Taipei, Taiwan; 2 Department of Materials Science and Engineering, National Chiao-Tung University, Hsinchu, 30010 Taiwan; 3 Biomedical Technology and Device Research Laboratories, Industrial Technology Research Institute, Chutung, Hsinchu 31040 Taiwan; 4 Department of Orthopedic Surgery, College of Medicine, National Taiwan University, No. 1, Ren-Ai Rd, Taipei, 10051 Taiwan, Republic of China; 5 Department of Orthopedic Surgery, National Taiwan University Hospital, No.7, Chung-Shan South Rd, Taipei, 10002 Taiwan, Republic of China; 6 Biomimetic Systems Research Center, National Chiao-Tung University, 1001 University Road, Hsinchu, 300 Taiwan, Republic of China; 7 School of Dentistry, College of Medicine, National Taiwan University, No 1 Chang-Te Street, Taipei, 10048 Taiwan; 8 Tissue Regeneration Product Technology Division, Biomedical Technology and Device Research Laboratories, Industrial Technology Research Institute, Hsinchu County, 310 Taiwan; 9 Industrial Technology Research Institute, Rm. 635, Bldg. 53, No. 195, Sec. 4, Chung Hsing Rd, Chutung, Hsinchu Taiwan

**Keywords:** Peri-implant osteomyelitis, Magnetic nanoparticle, Hyperthermia, Biofilm

## Abstract

**Background:**

When bacteria colony persist within a biofilm, suitable drugs are not yet available for the eradication of biofilm-producing bacteria. The aim of this study is to study the effect of magnetic nano-particles-induced hyperthermia on destroying biofilm and promoting bactericidal effects of antibiotics in the treatment of osteomyelitis.

**Methods:**

Sixty 12-weeks-old male Wistar rats were used. A metallic 18G needle was implanted into the bone marrow cavity of distal femur after the injection of Methicillin-sensitive *Staphylococcus aureus* (MSSA). All animals were divided into 5 different treatment modalities. The microbiological evaluation, scanning electron microscope examination, radiographic examination and then micro-CT evaluation of peri-implant bone resorption were analyzed.

**Results:**

The pathomorphological characteristics of biofilm formation were completed after 40-days induction of osteomyelitis. The inserted implants can be heated upto 75 °C by magnetic heating without any significant thermal damage on the surrounding tissue. We also demonstrated that systemic administration of vancomycin [VC (i.m.)] could not eradicate the bacteria; but, local administration of vancomycin into the femoral canal and the presence of magnetic nanoparticles hyperthermia did enhance the eradication of bacteria in a biofilm-based colony. In these two groups, the percent bone volume (BV/TV: %) was significantly higher than that of the positive control.

**Conclusions:**

For the treatment of chronic osteomyelitis, we developed a new modality to improve antibiotic efficacy; the protection effect of biofilms on bacteria could be destroyed by magnetic nanoparticles-induced hyperthermia and therapeutic effect of systemic antibiotics could be enhanced.

## Background

Infection of bone and soft tissue is one of the biggest problems in orthopedic daily practice, which mostly leads to progressive inflammation and even bone destruction [1]. Osteomyelitis is the infection and inflammation of bone and bone marrow tissue, usually begins as an acute infection, but it may evolve into a chronic condition. It can be subdivided into hematogenous or posttraumatic spread of bacteria, whereas the incidence of posttraumatic osteomyelitis is around 80% [2]. The treatment of chronic osteomyelitis is a challenging problem, since germs with variable drug sensitivities exist. The most common pathogens are coagulase negative staphylococci and *Staphylococcus aureus* [3]. There is still no evidence-based guidance on the treatment of chronic osteomyelitis and there is no single-treatment regimen or surgical procedure that is appropriate for all patients [2]. Besides the use of antibiotics, sufficient debridement seems to be the most important therapy for osteomyelitis [2].

In the presence of necrotic bone, a biofilm can develop and reduce the efficacy of antibiotic therapy by 10^3^ folds [4]. Bacteria may persist in a biofilm-based colony or be intra-cellularly concealed within osteoblasts, these characteristics render bacteria a greater resistance to the host’s immune response and antibiotic therapy [5]. Biofilm is any group of microorganisms in which cells stick to each other and adhere to a surface. These adherent cells are frequently embedded within a self-produced matrix of extracellular polymeric substance (EPS) [6]. Suitable drugs are not yet available for the eradication of such biofilm-producing bacteria.

Iron is an essential but potentially hazardous biometal. Mammalian cells require sufficient amounts of iron to satisfy metabolic needs or to accomplish specialized functions. Iron is an essential trace nutrient required for the active sites of many electron transfer and oxygen transport enzymes. In contrast, iron is also a catalyst for reactive oxygen species (ROS). Organisms must acquire iron but also must protect against oxidative damage. Ferritin is a naturally designed nanomachine for disposing cellular irons. Ferritin sequesters iron and maintains it in a biologically safe form. Ferritin also releases stored metals in response to cell needs. Thus, ferritin plays important roles in detoxification and reservation, and is vital for maintaining iron homeostasis in living cells. Ferritin genes are ubiquitously present among bacteria, plants, and animals, suggesting that they are essential genes for most organisms [7–9]. The iron oxide particle size of these agents varies widely, and influences their physicochemical and pharmacokinetic properties, and thus their clinical applications. Iron oxide nanocrystals (IONPs) are superparamagnetic. Many IONPs, including superparamagnetic iron oxides (SPIOs), are encapsulated by hydrophilic polymers to improve the stability and solubility. SPIOs have been used in clinical applications as a contrast agent for magnetic resonance imaging (MRI) [10, 11]. IONPs can be safely administered in the body because they do not raise concerns of heavy metal uptake and accumulation.

The healing effect of heat has been well-known since ancient times, it have been used to cure cancer [12] and infection [13]. The heating of biological tissue can be achieved by several physical mechanisms including microwave irradiation [14, 15], Ohmic heating [16], optical laser irradiation [17] or water bath heating [18, 19]. With these heating methods, it remains a challenge to control the spatial extent of heating in tissue. Magnetic particles hyperthermia improves the precision of heating by embedding the heating source into tissue and heating it using an external alternating magnetic field [20]. Many clinical trials have demonstrated the efficacy and safety of magnetic hyperthermia for prostate cancer and gliomas; patients can tolerate this therapy without discomfort or serious side effects [21–23]. However, there is no research for magnetic hyperthermia in the treatment of osteomyelitis. The aim of this study is to study the heating effect of magnetic nanoparticles-induced hyperthermia on destroying biofilm and promoting antibiotic efficacy for the treatment of peri-implant osteomyelitis.

## Mthods

### Synthesis of Fe_3_O_4_ magnetic nanoparticles

All chemicals, including 1,2-tetradecanediol, dibenzyl ether solution, oleic acid (C_17_H_33_COOH, technical grade 90%), oleylamine (C_18_H_35_NH_2_, technical grade 70%), Iron(III) acetylacetonate were obtained from (Fe(acac)_3_, Sigma-Aldrich, Inc., St. Louis, MO, USA). Fe_3_O_4_ magnetic nanoparticles were synthesized according to the following procedure. First, 2.0 mmole of Fe(acac)_3_, 10 mmole 1,2-tetradecanediol solution was added into 15 mL of dibenzyl ether solution, mixed with stabilizer and dispersant [containing 6 mmole oleic acid (C_17_H_33_COOH) and 6 mmole oleylamine (C_18_H_35_NH_2_)], stirring for 30 min at 130 °C and then then under a blanket of nitrogen, heated to reflux (290 °C) for 1 h. The dibenzyl ether which containing 0.03 M Fe^3+^ acetylacetonate mixture solution was added drop-wise under vigorous mechanical stirring (2000 rpm) for 30 min. The color of the suspension turned to black immediately. The suspension was cooled to room temperature by removing the heat source, ethanol (40 ml) was added to the mixture and a black material was precipitated and separated via centrifugation. The black product was dissolved in hexane in the presence of oleic acid (~0.05 mL) and oleyl amine (~0.05 mL). Centrifugation (6000 rpm, 10 min) was applied to remove any undispersed residue (almost none). The product, 6 nm Fe_3_O_4_ nanoparticles, was then precipitated with ethanol, centrifuged (6000 rpm, 10 min) to remove the solvent and redispersed into hexane. The precipitate was washed three times with doubly distilled water and then several times with ethanol. The supernatant solution was removed from the precipitate after decantation. Finally, the 6 nm Fe_3_O_4_ magnetic nanoparticles were dispersed with small amount of oleic acid (~0.05 mL) and oleylamine (~0.05 mL) and stored with hexane in a stoppered bottle for further use. The size and morphologies of products were characterized by transmission electron microscopy (TEM) of HITACHI TEM H-7500 microscope (HITACHI, Japan) with 200 mesh carbon film coated copper grid. The crystal structures of samples were recorded using a Rigaku X-ray powder diffractometer (Geigerflex; Tokyo, Japan).

### Alternating magnetic field (AMF) generator

An AMF was generated by a vertical coil with an inner diameter of 6.5 cm (Coil 1.5 A, 30 turns), driven by a transistor inverter (HOT SHOT; Ameritherm, New York, USA) operated at a frequency of 100 kHz and electric current (EC) 250 A which is capable of generating a 20G magnetic field. Thermal images were taken using a thermograph (infrared thermal imaging camera InfReC R300SR; Nippon Avionics, Tokyo, Japan). Temperature was also measured by using a thermograph.

The magnetic properties of Fe_3_O_4_ nanoparticles were analyzed by superconducting quantum interference device (SQUID, MPMS-XL7, Quantum Design, USA) at temperature 2 and 300 K with an applied field between −20 and 20 kOe; moreover, zero field cooling (ZFC) and field cooling (FC) measurement were carried out at 500 Oe to determine the blocking temperature (T_b_). The magnetic properties analyses were used to determine the blocking temperature (Tb), the temperature above which the magnetic property will change from ferrimagnetism to superparamagnetism, and magnetization curve which showed the saturation magnetization value for examining the feasibility of temperature increase within the AMF.

### In vitro hyperthermia effect

To evaluate the heating effect in vitro, the magnetic nanoparticles were placed in the bottom of a 1 mL eppendorf tube filled with 0.5 mL PBS. Each loaded eppendorf tube was set in the center of a two-turning copper coil (25 mm in diameter) in an alternating magnetic field (AMF, Power cube 64/900, ~ 750–1150 kHz, Ceia Co., Arezzo, Italy). Heat generation from the samples was determined by measuring the temperature change of PBS every 10 s with an optical fiber probe (STF-1 m) connected with the fluoroptic thermometer (I652, Luxtron, USA). The in vitro hyperthermia effect of the samples were carried out in the AMF as mentioned above and the eppendorf tubes were filled with 0.5 mL FBS. The heating process was performed for 30 s and the temperature was maintained at 74–75 °C by automatically switching an on/off device of the AMF.

### Animal model and surgical procedure

**Fig. 1:**
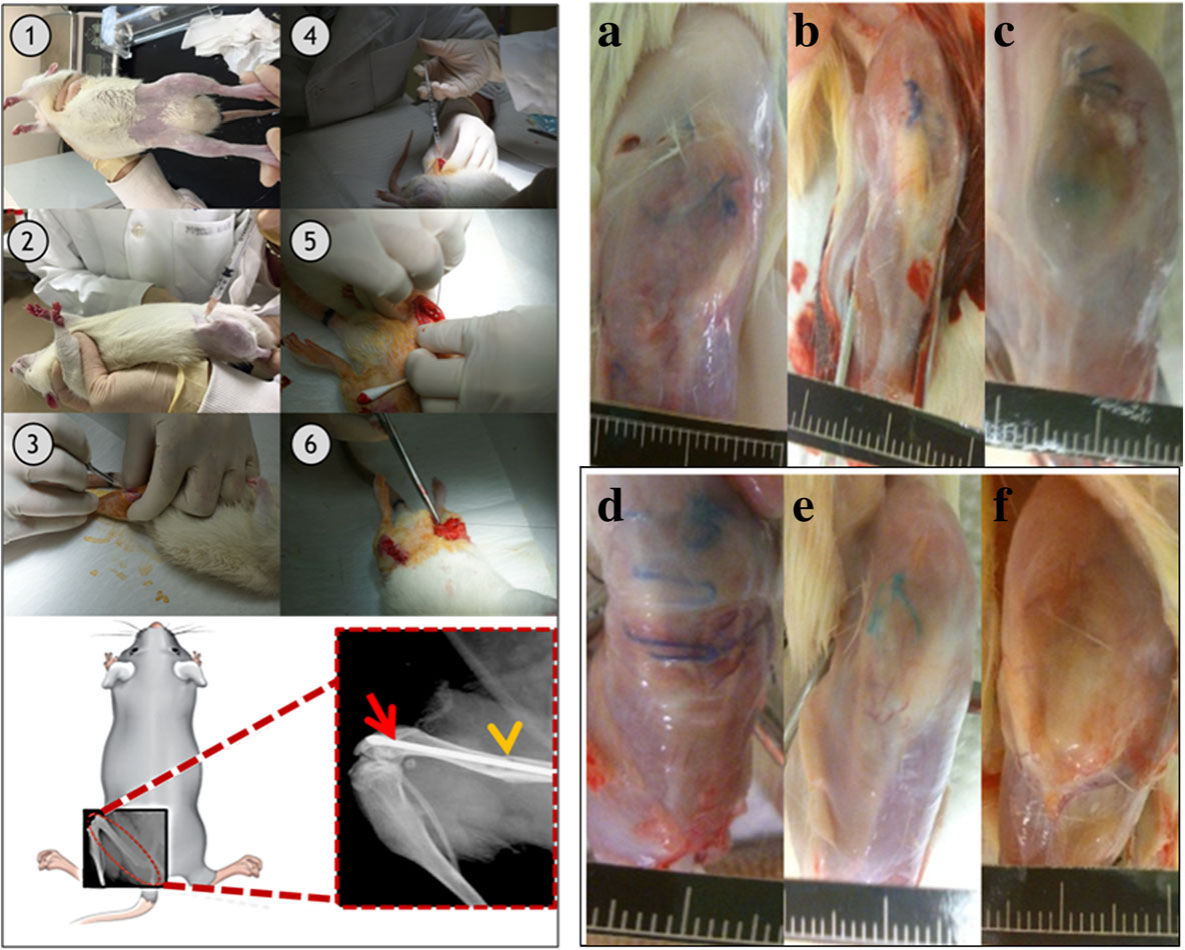
Sequence of surgical procedures and X-ray image and Animal models for induction of osteomyelitis. Left: Sequence of surgical procedures and X-ray image. Osteomyelitis animal models used 18G needle as implant and injecting methicillin-sensitive *Staphylococcus aureus* suspension into bone marrow canal as an infection model. The X-ray image showed that needle completely inserted into femur of rats. Right: Animal models for induction of osteomyelitis. **a** Uninfected group (left leg); (**b**) Uninfected group (right leg); (**c**) Osteomyelitis group (left leg): 10 days after induction of infection; (**d**) Osteomyelitis group (right leg): 10 days after induction of infection; (**e**) Osteomyelitis group (left leg): 40 days after induction of infection; (**f**) Osteomyelitis group (right leg): 40 days after induction of infection

The experimental protocol was approved by the Institutional Animal Care and Use Committee of Medical College, National Taiwan University (Taipei, Taiwan). Sixty male 12 weeks-old Wistar rats (300 ± 20 g were purchased from the Laboratory Animal Center, Medical College, National Taiwan University (Taipei, Taiwan) and acclimated under standard laboratory conditions at 22 ± 2 °C and 50 ± 10% humidity. Standard rat chow and water were available ad libitum during the acclimation period. Animals were anesthetized by Zoletil (15-18 mg/kg) and Rompun (0.05 ml/kg). Surgical interventions were performed under strict sterile conditions. The surgical area was shaved and disinfected with iodine, and a midline knee incision was made, followed by medial parapatellar arthrotomy and lateral patella subluxation to expose the knee joint. In the pilot study (24 rats), the osteomyelitis model was built by implanting metallic 18G needle into the bone marrow cavity of distal femur after injecting 10^5^ CFU/50 μl bacterial suspension of Methicillin-sensitive *Staphylococcus aureus* BCRC10451 (MSSA); while in the sham-operated negative control rats, metallic 18G needle was implanted without injection of MSSA (Fig. 1.). The strain used in this study, *S. aureus* BCRC10451 (MSSA), was cultivated overnight at 37 °C in brain heart infusion (BHI) broth (Sigma-Aldrich, Inc., St. Louis, MO, USA). The arthrotomy was closed using non-resorbable sutures in an interrupted pattern and then the skin was closed with resorbable suture. Intramuscular injection of cefazolie (20 mg/kg, Purzer Pharmaceutical Co., Ltd., Taipei, Taiwan) was used for postoperative prophylaxis. The formation of biofilm were confirmed by histological staining and scanning electron microscopy. In the pilot study, the rats were randomized to either 10, 20, 30 or 40 days endpoints to check the progression of histopathological change of osteomyelitis. Since the biofilm dormation was quite clear at 20 days after MSSA injection, we selected this time point for the following experiment to evaluate treatment efficacy of hyperthermia.

**Fig. 2:**
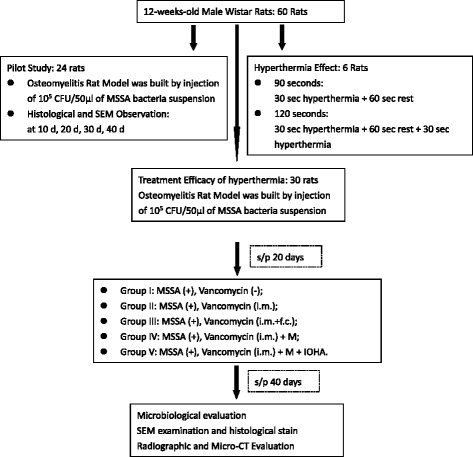
Flow Chart of experimental design

The evaluation for the treatment efficacy of magnetic hyperthermia, different treatment modalities (total volume 0.5 cm^3^) was implanted into the bone marrow cavity of distal femur by a metallic 23G needle. All of the 30 animals were divided into 5 groups (Fig. 2): Group I: osteomyelitis positive control without treatment; Group II: osteomyelitis treated with intramuscular injection of vancomycin [VC (20 mg/ kg, i.m., Sandoz Inc., Princeton, NJ, USA)]; Group III: osteomyelitis treated with both intramuscular and femur cavity injection of vancomycin [VC (20 mg/kg, i.m.) + VC (20 mg/ kg, f.c)]; Group IV: osteomyelitis treated with both intramuscular injection of vancomycin and magnetic particles [VC(20 mg/kg, i.m.) + M]; Group V: osteomyelitis treated with intramuscular injection of vancomycin and magnetic particles hyperthermia [VC(20 mg/kg, i.m.) + M + IOHA]. Animals from each group were sacrificed by overdose of pentobarbital at 40 days after surgery.

### Microbiological evaluation

Bacteriology swabs from the subcutaneous tissue and the implant site were obtained from all operation sites at the time of sacrifice. Swabs were moistened with one drop (20 μl) of sterile PBS, evenly streaked onto agar plate. The samples were incubated for 48 h at the 37 °C under anaerobic conditions. After 48 h’ incubation, the media were analyzed by conventional bacteriological techniques.

### Scanning electron microscope examination and histological staining

The morphology of biofilm was observed by scanning electron microscopy (SEM; Topcon ABT-60, Tokyo, Japan). Briefly, the specimens were fixed with 4% para-formaldehyde (PFA) for 2 h and 2% osmium tetroxide (OsO_4_) solution for 1 h, dehydrated in a graded series of ethanol, dried by critical-point drying (CPD) method, and sputter-coated with gold to a thickness film before observation.

For hematoxylin and eosin (HE) staining, randomly chosen femur from the osteomyelitis group was fixed in 5% formaldehyde for two days, decalcified by 5% nitric acid solution, and then embedded with paraffin. The tissues were cut and coronally sliced into 7 μm sections until use. Slices were stained with haematoxylin/eosin. Congo red staining and gram’s staining were also performed using the same sliced tissues. Six visual fields from each section were randomly selected and observed under light microscopy.

### Radiographic examination and micro-CT (computed tomography) evaluation

For radiographic evaluation, the soft tissue attached to the femur was remove, the rat femurs were examined by X-rays (Siemens, Germany). After radiologic examination, implants were removed and then assessed three dimensionally using Micro-CT (SkyScan 1176; Bruker Micro-CT, Kontich, Belgium) [24]. Datasets were reconstructed using CTvox 2.4 software for fast volumetric reconstruction, 2D/3D quantitative analysis and realistic 3D visualization. The tissue volume (TV: mm^3^), bone volume (BV: mm^3^) were recorded and then percent bone volume (BV/TV: %) was analyzed.

### Statistical analysis

All results were presented as means ± standard deviation (SD). Statistical analysis was performed for all the quantitative results using ANOVA test, the post hoc analysis used was Tukey’s honestly significant difference. The overall analysis was performed by the IBM Statistics SPSS 16.0 and the statistical significance in each test was set at *p* < 0.05.

## Results

**Fig. 3:**
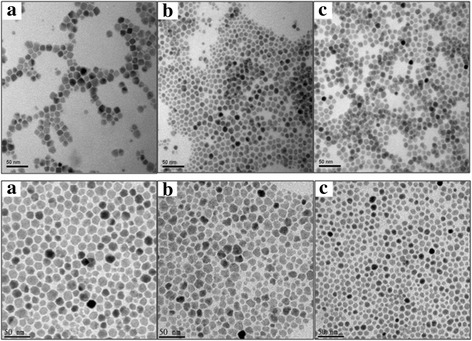
Transmission Electron Microscope (TEM) examination of Fe_3_O_4_ magnetic nanoparticles. Upper: TEM of F_3_O_4_ Nanoparticles: Effect of different amounts of stabilizer and dispersant. **a** 80 μL, **b** 160 μL, and **c** 400 μL surfactant. Lower: TEM of F_3_O_4_ Nanoparticles: Effect of different temperature on the formation F_3_O_4_ Nanoparticles. Injection temperature: **a** 260 °C, **b** 230 °C, and **c** 200 °C

Transmission electron microscope (TEM) examination of Fe_3_O_4_ magnetic nanoparticles was illustrated in the Fig. 3. In the presence of small amounts of stabilizer and dispersant, hydrophilic and stable Fe_3_O_4_ nanoparticles were synthesized (Fig. 3a); the formation of F_3_O_4_ nanoparticles did not significantly affected by the changes in the injection temperature (Fig. 3b).

**Fig. 4:**
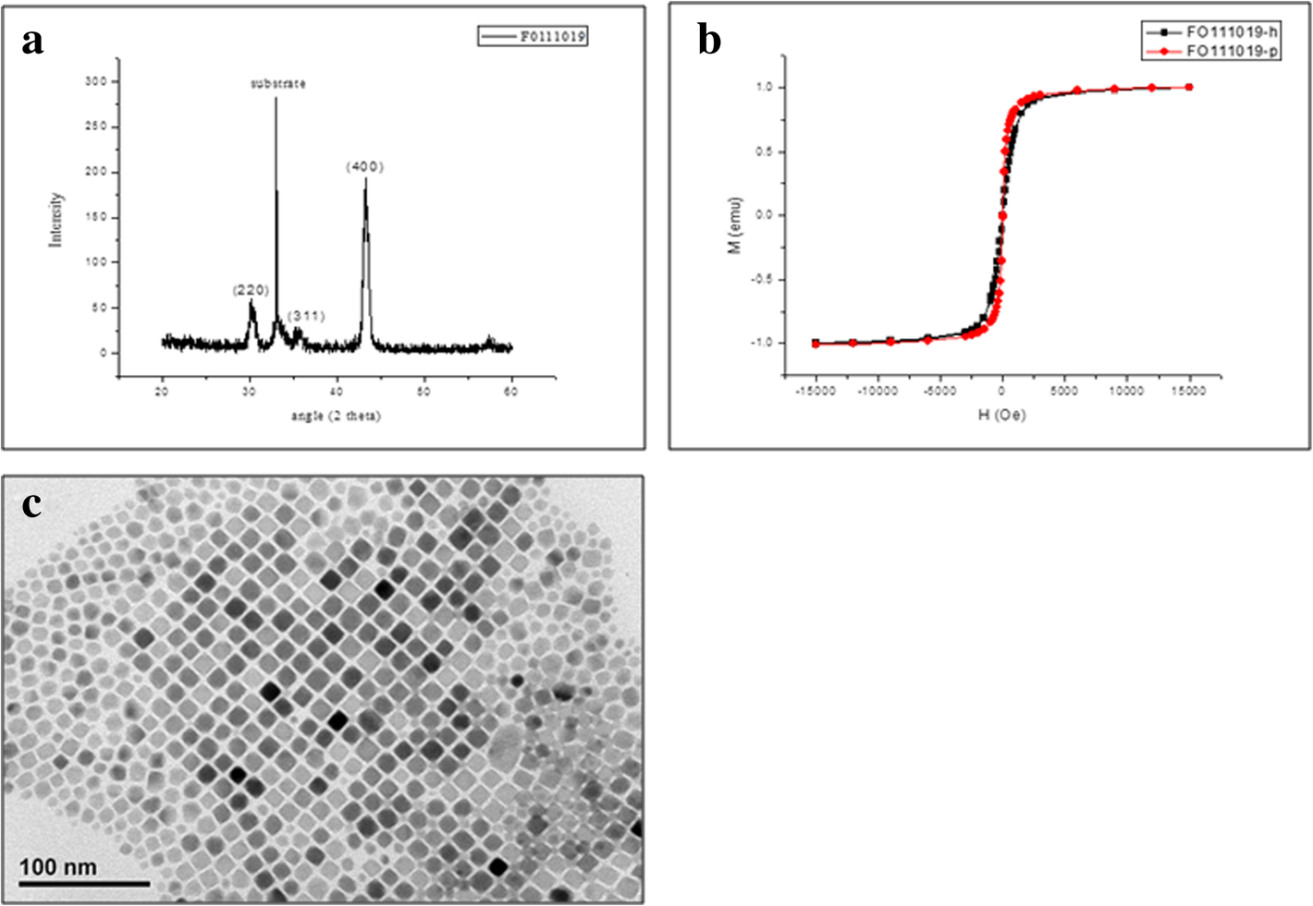
Characteristics of Fe_3_O_4_ nanoparticles. **a** The XRD pattern of the Fe_3_O_4_ nanoparticles. **b** Room-temperature magnetization curves for FO111019-P and FO111019-h. Magnetic hysteresis curves measured at room temperature for the samples prepared by a hydrothermal process (square: FO111019-h, dot: FO111019-p) showed the super-paramagnetic characteristics. **c** The crystal structure of Fe_3_O_4_ nanoparticles prepared at the injection temperature of 290 °C (TEM). The average diameter of particles is 16 nm

The crystal structure of Fe_3_O_4_ nanoparticles was confirmed by XRD spectroscopy. As shown in Fig. 4a, the diffraction peaks at 2θ = 30.4°, 35.5°, and 43.2° can be well indexed to (220), (311), and (400) planes of the inverse cubic spinel structure of Fe_3_O_4_ (JCPDS card no. 75–1610), respectively; according to the reflection peak positions and relative intensities, which confirms the formation of the Fe_3_O_4_ nanoparticles. The sample formed under a magnetic field is pure Fe_3_O_4_ without any impurity phases [1, 25]. Figure 4b was the hysteresis loops of Fe_3_O_4_ nanoparticles at room temperature. As shown in Fig. 4b, the hysteresis loops show that saturation magnetizations of Fe_3_O_4_ was 1.0 emu/g at the field of 1.5 KOe, and the absence of coercivity and remanence indicates that the as-prepared Fe_3_O_4_ has superparamagnetic properties above the blocking temperature. The TEM image in Fig. 4c shows that the average size of as-synthesized Fe_3_O_4_ magnetite nanoparticles is ca. 16 nm with narrow size distribution.

**Fig. 5:**
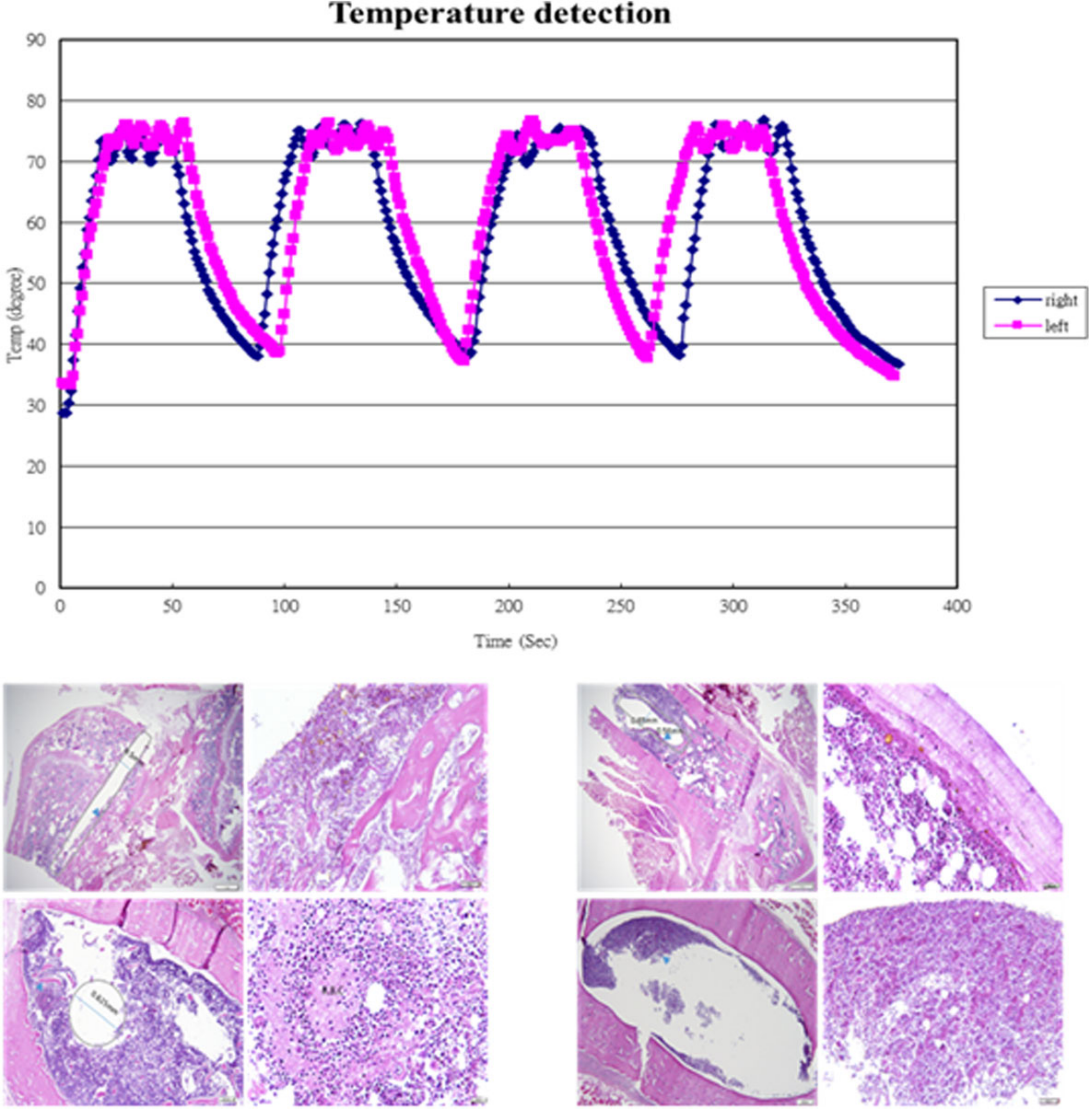
Tissue damage after thermotherapy (H & E staining). Left: The needle at 75 °C for 90 s by magnetic heating (each 30 s heating with a rest for 1 min), Right: The needle at 75 °C for 120 s by magnetic heating (each 30 s heating with a rest for 1 min). There were no significant thermal damage noted in these two testing conditions

Heat production is determined by the magnetic properties of Fe_3_O_4_ nanoparticles, its concentration, and the strength of the AMF. Therefore, we examined the heating effect of AMF on medium containing Fe_3_O_4_ nanoparticles by thermography. The temperature increased time-dependently, and we adjusted the required temperature dependent on the concentration of Fe_3_O_4_ nanoparticles and the magnitude of the electric current used to generate AMF to generate a temperature of 75 °C, and we adopted these conditions for the subsequent assays. In this study, the inserted implants can be heated to 75 °C by magnetic heating (Fig. 5). When the implants were heated to 75 °C either for 90 s or 120 s (each 30 s heating with a rest for 1 min), there were no significant thermal damage noted in these two testing conditions (Fig. 5).

**Fig. 6:**
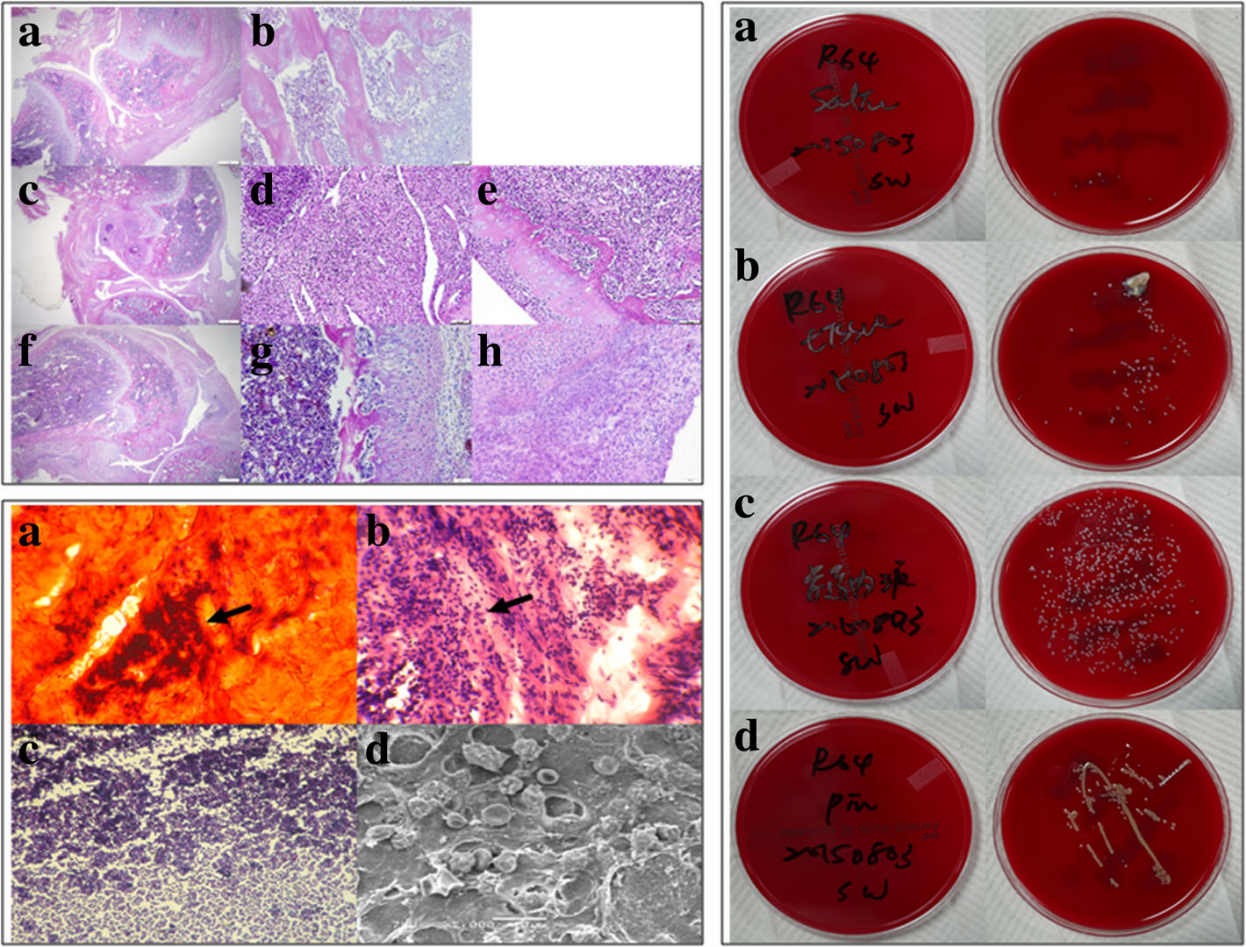
Histological, histopathological staining and microbiology assessment from osteomyelitis rats after 40 days infection. Left upper: Histological sections with H&E staining. Upper: Uninfected group, magnification **a** 40 × and **b** 1000 ×; Middle: Osteomyelitis 10 days after induction of infection, magnification **c** 40 ×, **d** and **e** 1000 ×; Lower: Osteomyelitis 40 days after induction of infection, magnification **f** 40 ×, **g** and **h** 1000 ×. In this figure, uninfected group possesses normal trabecular patterns of cancellous bone (upper: **a** and **b**); while in the acute suppurative changes of osteomyelitis, there is devitalized lamellar bone with scalloped edges and absence of stainable osteocytes and osteoblasts, edema and granulocytic infiltration of surrounding tissues are obvious (middle: **c**-**e**). In the chronic osteomyelitis, there is the irregular fragment of devitalized bone surrounded by dense fibrous tissue heavily infiltrated by plasma cells, lymphocytes, and only a few granulocytes (lower: **f**-**h**). *Left lower: Histopathological staining.* **a** Cango red staining, **b** H&E staining, **c** Gram staining and **d** SEM morphology, respectively. As shown here, GRAM-staining revealed the presence of bacteria within the defect site as Gram (+) *Staphylococcus aureus* bacteria could be detected (**b** and **c**). Cango red staining (**a**) and SEM image (**d**) showed the biofilm forming completed after 40 days induction of osteomyelitis. Magnification of pictures are 1000 ×. *Right: Microbiology assessment from osteomyelitis rats after 40 days infection.* Specimens streaked onto agar plate for: **a** Sterilized PBS as control group; **b** Tissue from the affected area; **c** Intramedullary liquid from the affected femur; **d** Implants from the affected animals. All smears taken from the experimental sites showed characteristic accretion of *S. aureus* infection. In the control group, there is few characteristic CFUs on the agar plate (**a**). On the other hand, tissue from the affected area, intramedullary liquid from the infected femur and the implants from the infected animals all showed characteristic growth of CFUs on agar plates (**b**-**d**)

All animals survived after the surgical procedures and no postoperative skin infection or fever was observed. Figure 6 showed the histological, histopathological staining and microbiology assessment from osteomyelitis rats after 40 days infection (Fig. 6. Left upper). In this figure, uninfected group possesses normal trabecular patterns of cancellous bone (upper: Fig. 6 Left upper: A and B); while in the acute suppurative changes of osteomyelitis, there was devitalized lamellar bone with scalloped edges and absence of stainable osteocytes and osteoblasts, edema and granulocytic infiltration of surrounding tissues are obvious (middle: Fig. 6 Left upper: C-E). In the chronic osteomyelitis, there was the irregular fragment of devitalized bone surrounded by dense fibrous tissue heavily infiltrated by plasma cells, lymphocytes, and only a few granulocytes (lower: Fig. 6 Left upper: F-H).

In the histopathological staining, we verified the pathomorphological characteristics of our infected rat femur (Fig. 6 Left lower). As shown here, GRAM-staining revealed the presence of bacteria within the defect site as Gram (+) *Staphylococcus aureus* bacteria could be detected (Fig. 6 Left lower: B and C). Cango red staining (Fig. 6 Left lower: A) and SEM image (Fig. 6 Left lower: D) showed the biofilm forming completed after 40 days induction of osteomyelitis.

All smears taken from the experimental sites showed characteristic accretion of *S. aureus* infection as depicted in Fig. 3. In the control group, there is few characteristic CFUs on the agar plate (Fig. 6 Right: A). On the other hand, tissue from the affected area, intramedullary liquid from the infected femur and the implants from the infected animals all showed characteristic growth of CFUs on agar plates (Fig. 6 Right: B-D).

**Fig. 7:**
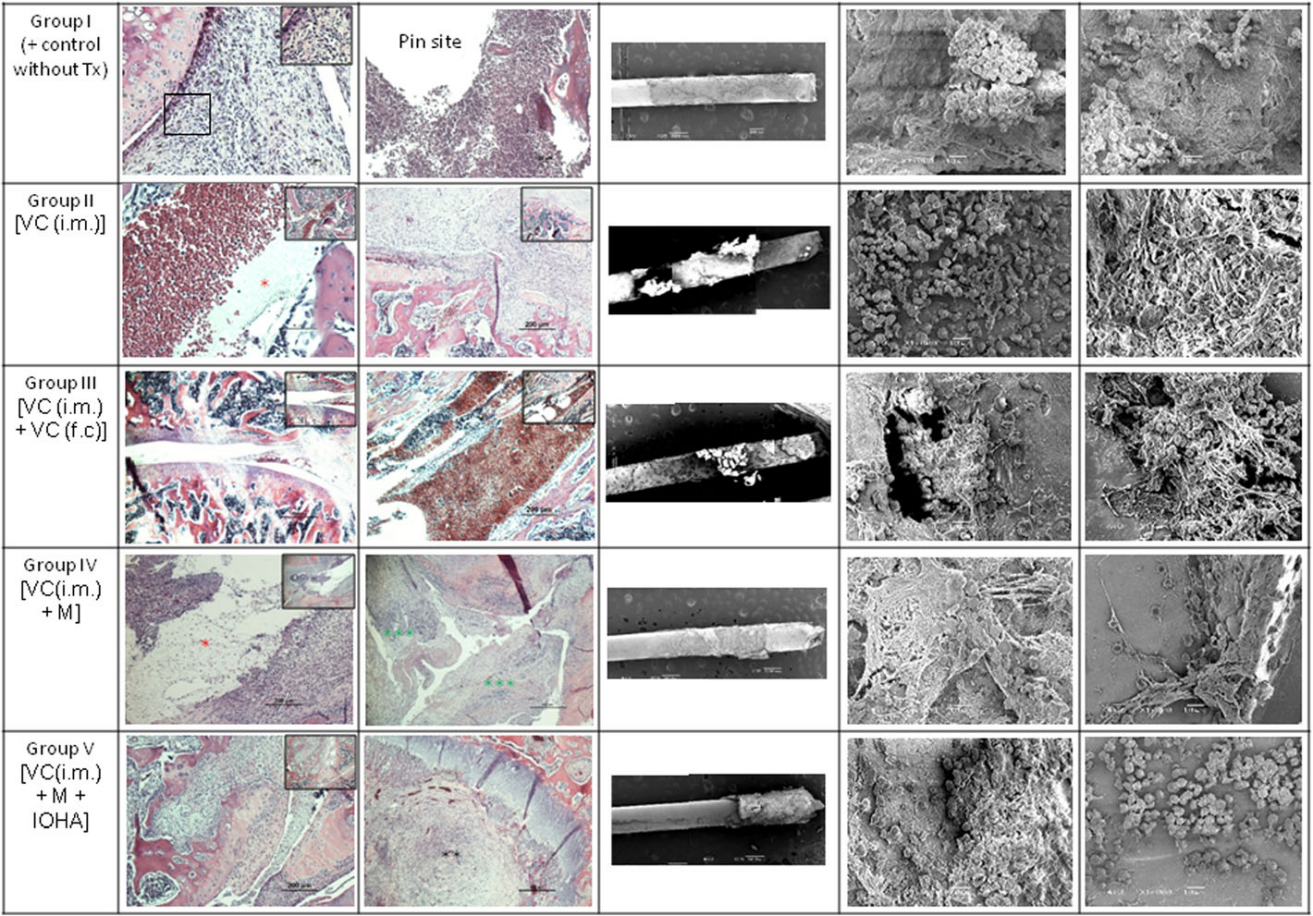
Histological and scanning electron microscope examination of different treatment modalities for osteomyelitis rats after 40 days infection. After inoculation of *Staphylococcus aureus*, tissue necrosis with active inflammation and even biofilm formation was clearly demonstrated in all groups; while, the biofilm formation were found both the at the surface of the inserted pin and the inner surface of bony trabeculae. Group I: osteomyelitis positive control without treatment; Group II: osteomyelitis treated with intramuscular injection of vancomycin [VC (i.m.)]; Group III: osteomyelitis treated with both intramuscular and femur cavity injection of vancomycin [VC (i.m.) + VC (f.c)]; Group IV: osteomyelitis treated with both intramuscular injection of vancomycin and magnetic particles [VC(i.m.) + M]; Group V: osteomyelitis treated with intramuscular injection of vancomycin and magnetic particles hyperthermia [VC(i.m.) + M + IOHA]. Animals from each group were sacrificed by overdose of pentobarbital at 40 days after surgery. *(red): biofilm-like ECM; **(*black*): necrosis with inflammation; ***(green): inflammation

After the inoculation of *Staphylococcus aureus*, tissue necrosis with active inflammation and even biofilm formation were clearly demonstrated in all groups; while, the biofilm formation were found both at the surface of the inserted pin and the inner surface of bony trabeculae. Different treatment modalities did not change the histopathological features of the induced osteomyelitis; while massive necrosis of tissue was observed in the group V which was treated with intramuscular injection of vancomycin and magnetic particles hyperthermia [VC(i.m.) + M + IOHA] (Fig. 7).

**Fig. 8:**
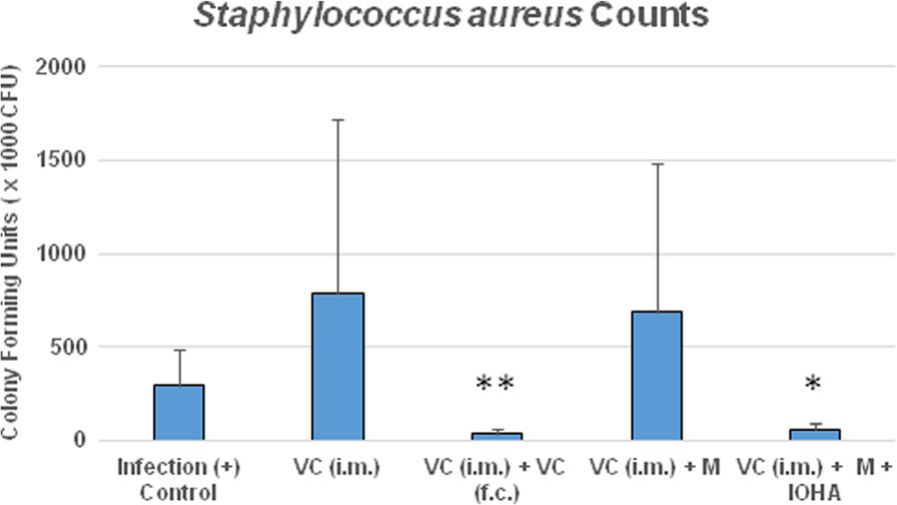
Colony-forming unit of implants and the affected area under different treatment modalities. Systemic administration of vancomycin [VC (i.m.)] did not show any better effect on eradication of the bacteria; similar event observed in the presence of magnetic nanoparticles [VC (i.m.) + M]. Local administration of vancimycin into the femoral canal [VC (i.m.) + VC (f.c)] did enhance the eradication of bacteria; similar event observed in the presence of magnetic nanoparticles when magnetic field was applied [VC (i.m.) + M + IOHA]

In our study, we demonstrated that systemic administration of vancomycin [VC (i.m.)] did not show any effect on eradication of the bacteria when compared with the positive control group; similar event observed in the presence of magnetic nanoparticles [VC (i.m.) + M]. But, local administration of vancomycin into the femoral canal [VC (i.m.) + VC (f.c)] did enhance the eradication of bacteria; similar event was observed in the presence of magnetic nanoparticles when magnetic field was applied [VC (i.m.) + M + IOHA] (Fig. 8).

**Fig. 9:**
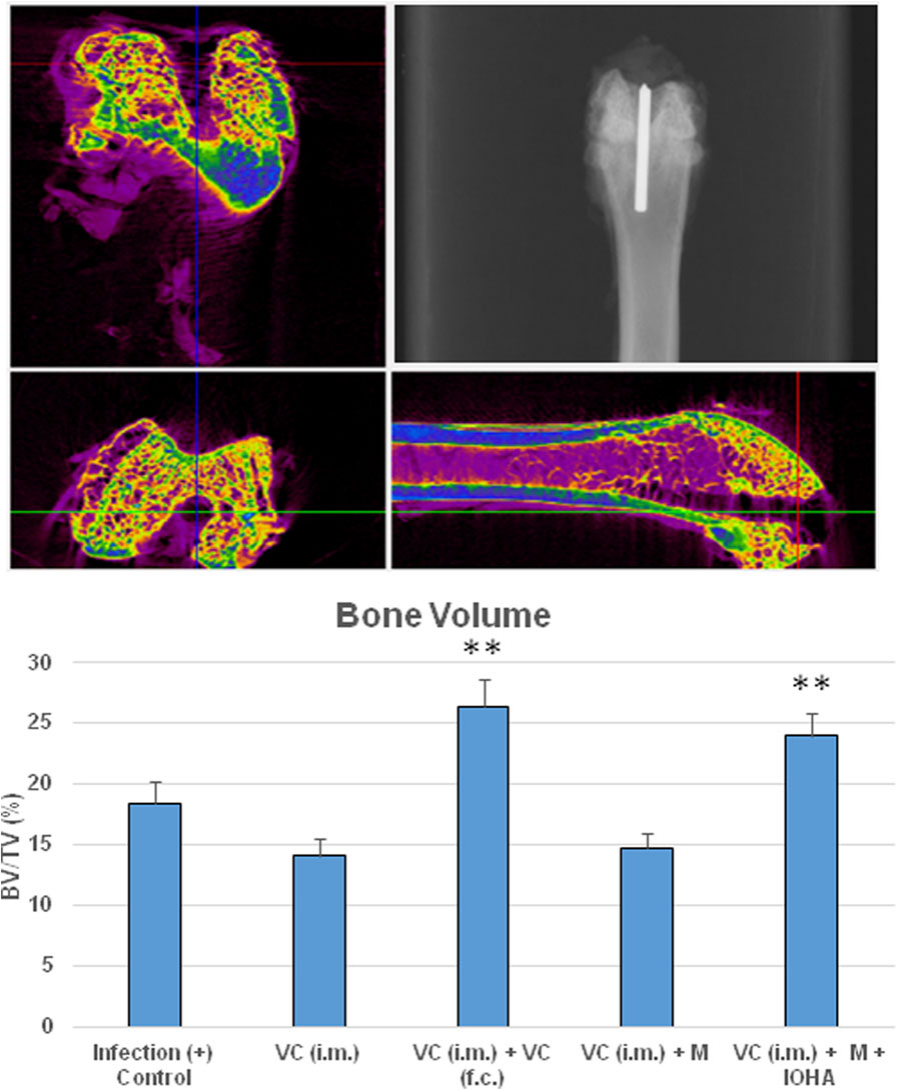
Change of relative bone volume at the affected area under different treatment modalities. Upper: Micro-CT (Computed Tomography) evaluation and radiographic examination. Lower: Systemic administration of vancomycin [VC (i.m.)] with/or without the presence of magnetic nanoparticles [VC (i.m.) + M] did not affect the bone volume change under peri-implent osteomyelitis condition; but, percent of bone volume (BV/TV) was significantly higher in the group of local administration of vancomycin into the femoral canal [VC (i.m.) + VC (f.c)] and the group when magnetic field was applied in the presence of magnetic nanoparticles [VC (i.m.) + M + IOHA]

In the peri-implant osteomyelitis, systemic administration of vancomycin [VC (i.m.)] with/or without the presence of magnetic nanoparticles [VC (i.m.) + M] did not affect the percent bone volume; but, when local administration of vancomycin into the femoral canal [VC (i.m.) + VC (f.c)] or magnetic field was applied in the presence of magnetic nanoparticles [VC (i.m.) + M + IOHA]. The percent bone volume (BV/TV) was significantly higher than that of the positive control (Fig. 9).

## Discussion

Currently, there are no evidence-based guidelines in terms of the treatment of chronic osteomyelitis [2]. It is difficult to achieve complete remission, some studies report a failure rates of 20–60% [26]. The aim of treatment is to improve quality of life through either a curative or a palliative strategy. Curative management strategies usually comprise of a combination of complex surgical procedures and tailored adjuvant antibiotic therapy [27]; on the other hand, palliative treatment strategies are less invasive and typically involve to the use of chronic suppressive antibiotic therapy [28].

Recognizing the importance of the host’s physiological status during formulation of a treatment plan, Cierny and Mader revolutionized the approach to chronic osteomyelitis through the publication of their clinical staging system in 1985 (Table 1) [29]. In our model, there is seriously immune response in bone marrow and synovial fluid. Our results exhibit that, after *S. aureus* bacterial inoculation and incubation, a steady infection in femur was generated (Fig. 6 Left upper). According to this classification system, we created a localized type of osteomyelitis in hosts with uncompromised immune systems, corresponding to type IIIA osteomyelitis. In the histopathological staining, we verified the pathomorphological characteristics of our infected rat femur and the biofilm formation was completed after 40 days induction of osteomyelitis (Fig. 6 Left lower), with characteristic accretion of *Staphylococcus aureus* infection presents at different tissues obtained from the affected area (Fig. 6 Right).

**Table 1:** Cierny and Mader clinical staging system for adult chronic osteomyelitis^**28**^

Anatomic type	
I	Medullary osteomyelitis
II	Superficial osteomyelitis
III	Localized osteomyelitis
IV	Diffuse osteomyelitis
Physiological Class	
A	Good immune system and delivery
B	Compromised locally (BL) or systemically (BS)
C	Requires suppressive or no treatment; minimal disability; treatment worse than disease; not a surgical candidate
Clinical Stage	
	Type + Class = Clinical stage

Hyperthermia (HT) is a minimally invasive method for cancer therapy in which the target tissues are annealed to about 42–46 °C. Heat treatment in such a temperature range is cytotoxic for tumor cells because of their disorganized and compact vascular structure that promotes an unfavorable microenvironment inside them. Although HT is a promising approach to cancer therapy, the inevitable technical problem with hyperthermia is the difficulty in heating only the local tumor region to the intended temperature without damaging the surrounding healthy tissue [30]. Magnetic nanoparticles are increasingly used for clinical applications such as drug delivery, magnetic resonance imaging and magnetic fluid hyperthermia. Magnetic nanoparticles (MNPs) have been used for HT treatment in an attempt to overcome this obstacle [23, 31]. The magnetic nanoparticles have been focus on many research recently because they possess attractive properties which could be potential used in biomedicine, tissue specific targeting, magnetic resonance imaging, defect sensor and environmental remediation, etc. [32–36].

In this study, the inserted implants can be heated to 75 °C by magnetic heating; while, there were no significant thermal damage noted in this testing condition (Fig. 5) Due to magnetic particles had better dispersion, magnetic nanoparticles (MNPs) heating sources was related to the better distribution of heating intensity in tissue [37, 38]. We did observed that after inoculation of *Staphylococcus aureus*, tissue necrosis with active inflammation and even biofilm formation were clearly demonstrated in all groups; the biofilm formation were found both the at the surface of the inserted pin and the inner surface of bony trabeculae. Different treatment modalities did not change the histopathological features of the induced osteomyelitis; while massive necrosis of tissue was observed in the group V which was treated with intramuscular injection of vancomycin and magnetic particles hyperthermia [VC(i.m.) + M + IOHA] (Fig. 7). As shown above, since this parameter did not cause tissue damage, we implied that the induced hyperthermia can enhance the destruction of *Staphylococcus aureus*.

In this study, rat osteomyelitis model was built by implanting metallic needle into the marrow cavity of distal femur after injecting 10^8^ CFU/0.5 ml of bacterial suspension. In this model, intramuscular injection of vancomycin [VC (i.m.)] did not show any effect to treat osteomyelitis. In the group of intramuscular injection of vancomycin with magnetic environment also could not make biofilm collapse and even to enhance bacteria growth [VC (i.m.) + M]. By raising the temperature of implant, adding magnetic nanoparticles to support hyperthermia that could destroy biofilm and enhance efficacy of vancomycin significantly [VC (i.m.) + M + IOHA] (Fig. 8). In the peri-implant osteomyelitis, local administration of vancomycin into the femoral canal [VC (i.m.) + VC (f.c)] or magnetic field application in the presence of magnetic nanoparticles [VC (i.m.) + M + IOHA] did prevent the bone loss associated with infection (Fig. 9).

A number of animal models were created in order to study the treatment effects of bacterial infections. *Staphylococcus aureus* strain for bacterial inoculation was used in almost all of them, because this is the most common pathogen in the setting of osteomyelitis [3, 39]. Furthermore, rats and rabbits were used in most studies because they are big enough to create stable long bone infections without giant efforts; while on the other hand they are small enough to be housed and handled easily [40–43]. Bone and intramedullary bacterial infections are one of the most serious complications of the surgical repair of fractures. Treatment of implant-related chronic osteomyelitis is often difficult and usually consists of implant removal, extensive surgical debridement, and prolonged antibiotic use. To reduce the incidence of implant-related infections, several biomaterial surface treatments with integrated antibiotics, antiseptics, or metal ions have been developed for implants to reduce implant-related infections [44, 45]. In the current study, we developed a rat model for studies related to improve the treatment of chronic osteomyelitis.

Biofilms are universal, occurring in aquatic and industrial water systems as well as a large number of environments and medical devices relevant for public health. Recent clinical research has implicated biofilms in the exacerbation of wounds [46, 47]. Biofilm formation, which decrease activity of antibiotics, is a crucial step in the pathogenesis of many subacute and chronic bacterial infections, including infection after artificial joints surgery. In this study, we developed a novel antimicrobial thermotherapy platform to treat chronic osteomyelitis. Previous studies always focused on a successful application of hyperthermia in cancer therapy [48, 49]. However, to our knowledge, this is the first in-vivo application of thermotherapy for osteomyelitis. Because of differences in the temperature sensitivity of *Staphylococcus aureus* compared to cancer cells, transient higher temperature must be safe for realization of this technology as an effective therapeutic adjuvant for infectious pathogens. Our result showed temperature up to 75 °C is still not causing tissue damage. More important, thermotherapy may be successfully combined with standard-of-care antibiotic treatment for polymicrobial biofilm infections and enhance antibiotic penetration by disrupting the biofilm polysaccharide matrix. This may significantly enhance the efficacy of a given antimicrobial effect and facilitate reduced drug dose, thus providing a valuable tool for the treatment of fragile patients who cannot tolerate high dose systemic antibiotics.

## Conclusion

In this study, we use the needle implantation and injection of methicillin-sensitive *Staphylococcus aureus* bacterial suspension to create an animal model of osteomyelitis which mimic the clinical situation as chronic infection after implantation of artificial materials. Due to biofilms decreased the efficiency of antibiotics, using systemic antibiotic only to treat osteomyelitis did not show therapeutic difference with that of the positive control group. Here, we developed a new treatment modality to improve antibiotic efficacy in the treatment of chronic osteomyelitis. As the smaller size of magnetic naoparticles and their better dispersion in the infected surroundings, nanoparicles-induced hyperthermia can be evenly distributed to the whole surface of biofilm; the biofilms could be destroyed by magnetic nanoparticles hyperthermia and therapeutic effect of systemic antibiotics could be enhanced.

## Abbreviations

BV/TV: percent bone volume
CPD: critical-point drying
EPS: extracellular polymeric substance
f.c: femur cavity injection
HE staining: hematoxylin and eosin staining
IOHA: magnetic particles hyperthermia
IONPs: iron oxide nanocrystals
M: magnetic particles
Micro-CT: Micro-Computed Tomography
MSSA: methicillin-sensitive *Staphylococcus aureus*
PFA: para-formaldehyde
ROS: reactive oxygen species
s.c: subcutaneous injection
SPIOs: superparamagnetic iron oxides
TV: tissue volume
VC: vancomycin
